# Mechanisms and Functions of Mitophagy and Potential Roles in Renal Disease

**DOI:** 10.3389/fphys.2020.00935

**Published:** 2020-08-07

**Authors:** Zhenying Zuo, Kaipeng Jing, Hongluan Wu, Shujun Wang, Lin Ye, Zhihang Li, Chen Yang, Qingjun Pan, Wei Jing Liu, Hua-feng Liu

**Affiliations:** ^1^ Key Laboratory of Prevention and Management of Chronic Kidney Disease of Zhanjiang City, Affiliated Hospital of Guangdong Medical University, Zhanjiang, China; ^2^ Institute of Nephrology, Affiliated Hospital of Guangdong Medical University, Zhanjiang, China; ^3^ Key Laboratory of Chinese Internal Medicine, Ministry of Education and Beijing, Dongzhimen Hospital Affiliated to Beijing University of Chinese Medicine, Beijing, China; ^4^ Renal Research Institution of Beijing University of Chinese Medicine, Dongzhimen Hospital Affiliated to Beijing University of Chinese Medicine, Beijing, China

**Keywords:** mitophagy, mitochondrial dysfunction, acute kidney injury, diabetic kidney disease, lupus nephritis

## Abstract

Mitophagy is an evolutionarily conserved process to selectively remove damaged or unnecessary mitochondria *via* the autophagic machinery. In this review, we focus on recent advances in the molecular mechanisms of mitophagy and how mitophagy contributes to cellular homeostasis in physiological and pathological contexts. We also briefly review and discuss the crosstalk between mitophagy and renal disease, highlighting its modulation as a potentially effective therapeutic strategy to treat kidney diseases such as acute kidney injury (AKI), diabetic kidney disease (DKD), and lupus nephritis (LN).

## Introduction

Mitochondria are essential organelles that regulate cellular metabolism, homeostasis, and stress responses. Mitophagy, a process by which dysfunctional or superfluous mitochondria are selectively eliminated by autophagy, is the central mechanism of mitochondrial quality and quantity control (Pickles et al., 2018). Recent progress in studies of mitophagy has revealed that mitochondrial priming is mediated by the phosphatase and tensin homolog (PTEN)-induced kinase 1 (PINK1)/Parkin signaling pathway and/or by mitophagy receptors (Palikaras et al., 2018). Defective mitophagy has been implicated in various human diseases, such as aging, neurodegenerative disease, cardiovascular disease, and cancer (Song et al., 2015; Shi et al., 2018; Kingwell, 2019; Vara-Perez et al., 2019). The kidney is an energetically demanding organ and is rich in mitochondria. Further, renal function is highly dependent on mitophagy (Sun et al., 2015). Emerging evidence suggests that aberrant or defective mitophagy is central to the pathology of many renal diseases (Wang et al., 2020b). Here, we review recent progress in delineating the molecular mechanisms of mitophagy and highlight the specific effects of mitophagy on renal function and the possible cooperation between distinct mechanisms of mitophagy. We further discuss the association between mitophagy and the pathogenesis of renal diseases, including acute kidney injury (AKI), diabetic kidney disease (DKD), and lupus nephritis (LN).

## Mitophagy Overview

### Mitochondrial Structure and Function

Mitochondria are double-membrane-bound organelles located in the cytoplasm of nearly all eukaryotic cells. Mitochondria consist of an intermembrane space and mitochondrial matrix separated by the outer mitochondrial membrane (OMM) and inner mitochondrial membrane (IMM). Mitochondria are assembled through interplay between the nuclear and mitochondrial genomes. Mammalian mitochondrial DNA (mtDNA) encodes 37 genes, 13 of which encode polypeptide components of the oxidative phosphorylation machinery, in addition to the 22 tRNAs and two rRNAs required for gene transcription and translation within the organelle (Barshad et al., 2018; Murphy and Hartley, 2018). Approximately 99% of mitochondrial proteins are encoded by nuclear genes, translated on cytoplasmic ribosomes, and imported into mitochondria by the translocase of the outer membrane (TOM) and translocase of the inner membrane (TIM) complexes (Pfanner et al., 2019). The core function of mitochondria is energy metabolism, and mitochondria are considered to be the cellular energetic powerhouse (Murphy and Hartley, 2018). In addition to energy metabolism, mitochondria are involved in fatty acid synthesis, amino acid production, heme synthesis, and iron-sulfur cluster biogenesis (Murphy and Hartley, 2018). Mitochondria also communicate with the endoplasmic reticulum (ER) *via* the mitochondrial-associated ER membrane (MAM) to regulate Ca^2+^ and lipid homeostasis, as well as apoptosis (Lee and Min, 2018). In addition, mitochondria are a major site of reactive oxygen species (mtROS) production. Under normal conditions, mtROS are quickly scavenged by superoxide dismutase 2 (SOD2) in the mitochondrial matrix and SOD1 in the mitochondrial intermembrane space, as well as other antioxidant enzymes, including glutathione peroxidase (GPX), glutathione (GSH), and glutathione disulfide (GSSG; Li et al., 2013; Wang and Tabas, 2014). Physiological levels of mtROS are essential for various signaling functions and maintain the functional state of mitochondria (Sena and Chandel, 2012), while excessive mtROS cause oxidative damage to proteins, lipids, and DNA, leading to a adenosine triphosphate (ATP) depletion and further increases of mtROS production, as well as inflammasome activation, which exacerbates cellular damage and initiates programmed cell death (Boengler et al., 2017). Hence, timely elimination of damaged and aged mitochondria is essential for maintaining cellular integrity (Pickles et al., 2018).

### Molecular Mechanisms of Mitophagy

**Figure 1:**
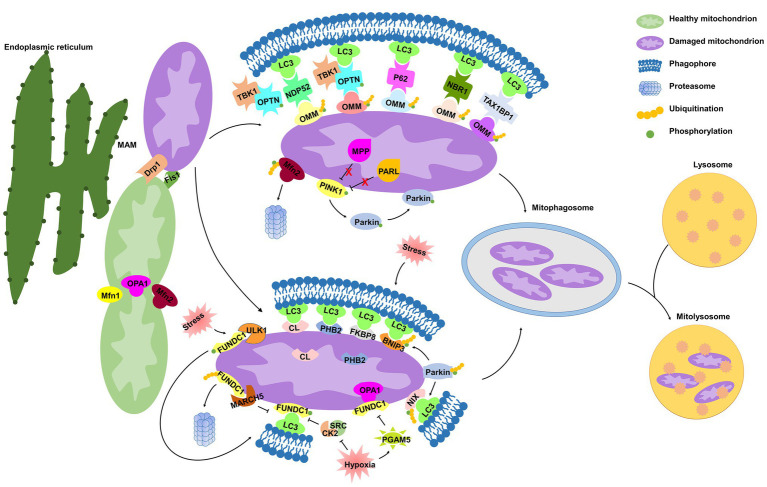
Molecular mechanisms of mitophagy. Under cell stress, dynamin-related protein 1 (Drp1) is recruited from the cytoplasm to mitochondria at the mitochondrial-associated endoplasmic reticulum (ER) membrane (MAM), while mitochondrial fusion regulated by mitofusin 1 (Mfn1), mitofusin 2 (Mfn2), and optic atrophy 1 (OPA1) is inhibited, both of which dissociate mitochondria from the ER and collaboratively mediate mitochondrial fragmentation, initiating the process of mitophagy. In damaged mitochondria, the proteolytic degradation of phosphatase and tensin homolog (PTEN)-induced kinase 1 (PINK1) by the matrix protein mitochondrial processing peptidase (MPP) and by the inner mitochondrial membrane (IMM) protein presenilin-associated rhomboid-like protease (PARL) is halted, and thus PINK1 is stabilized on the outer mitochondrial membrane (OMM), where it phosphorylates both Parkin and ubiquitin. Parkin is then recruited to mitochondria and contributes to ubiquitination of several OMM proteins that function as “eat-me” signals. Adaptor proteins [p62, optineurin (OPTN), NDP52, NBR1, and TAX1BP1] recognize phosphorylated poly-ubiquitin chains on OMM proteins and initiate mitophagosome formation through binding with microtubule-associated protein 1 light chain 3 (LC3). In addition, IMM proteins [cardiolipin (CL) and prohibitin 2 (PHB2)] are externalized to OMM following mitochondrial damage. In response to different stimuli, CL, PHB2, and other mitophagy receptors [FUN14 domain-containing protein 1 (FUNDC1), NIX, BCL2 interacting protein 3 (BNIP3), and FKBP prolyl isomerase 8 (FKBP8)] interact directly with LC3. Once mitophagosome is formed, it fuses with lysosome to generate mitolysosome, mediating mitochondrial elimination.

Mitophagy, a term first proposed by Lemasters (2005), refers to a process by which mitochondria are selectively degraded by autophagy (Palikaras et al., 2018), which has emerged as an important mechanism of mitochondrial quality and quantity control (Pickles et al., 2018). Mitophagy is a mechanistically elaborate process including (a) mitophagy initiation, (b) priming of mitochondria for recognition by autophagy machinery, (c) engulfment of the marked mitochondria by formation of the autophagosome, and (d) lysosomal sequestration and hydrolytic degradation (Figure 1; Vasquez-Trincado et al., 2016). Several regulatory pathways of mitophagy are classified into PINK1/Parkin-mediated mitophagy and receptor-mediated mitophagy and are initiated in response to different stimuli (Gkikas et al., 2018).

#### Role of Mitochondrial Dynamics in Priming Mitochondria for Mitophagy

The machinery regulating mitochondrial dynamics is highly integrated with initiation of mitophagy (Twig and Shirihai, 2011). Mitochondria are highly dynamic organelles engaged in repeated cycles of fusion and fission, which maintain mitochondrial integrity by facilitating mtDNA and protein quality control (Detmer and Chan, 2007). Mitochondrial dynamics is regulated by members of a family of conserved large GTPases. In mammals, cytoplasmic dynamin-related protein 1 (Drp1) and the OMM protein Fis1 mediate mitochondrial fission, whereas mitofusins 1 and 2 (Mfn1 and Mfn2) mediate OMM fusion, and optic atrophy 1 (OPA1) mediates IMM fusion (Detmer and Chan, 2007).

Surprisingly, MAM is also related to mitochondrial dynamics, defining the sites of mitochondrial division (Friedman et al., 2011; Wu et al., 2018). The attachment of mitochondria to the ER depends on Mfn2 (Basso et al., 2018). Under cell stress, Drp1 is recruited from the cytoplasm to mitochondria at the MAM (Wu et al., 2018), and Mfn2 is ubiquitinated in a p97-dependent manner (McLelland et al., 2018), both of which dissociate mitochondria from the ER and collaboratively mediate mitochondrial fragmentation (McLelland et al., 2018; Fonseca et al., 2019). Upon fission, mitochondria can be segregated into polarized daughter mitochondria, exhibiting a high probability of subsequent fusion, and depolarized mitochondria are specifically targeted for mitophagic elimination (Twig et al., 2008). Lee et al. (2011) demonstrated that Drp1 inhibition prevents the BCL2 interacting protein 3 (BNIP3)-mediated mitophagy in cultured adult cardiac myocytes in basal conditions. These studies suggest that mitochondrial fragmentation is a prerequisite for mitophagy.

Notably, recent *in vivo* and *in vitro* studies have revealed a higher level of complexity in mitophagy. In a conditional cardiac *Drp1*-deficient mouse model, *Drp1* knockout promotes mitochondrial enlargement, dilated cardiomyopathy, and necrotic cell death and increases mitophagy that is associated with mitochondrial permeability transition pore opening (Song et al., 2015). Conversely, conditional *Mfn1/2* deletion provokes accumulation of fragmented and dysfunctional mitochondria, without interfering with mitophagy (Song et al., 2015). Another study, however, demonstrated that Mfn2 promotes Parkin translocation and phosphorylation to trigger mitophagy, mechanistically linking mitochondrial fusion with mitophagy that coordinately maintains mitochondrial quality (Xiong et al., 2019). Hence, we should warrant a re-evaluation of the assumption that mitochondrial fragmentation is a prerequisite for mitophagy. It seems that mitochondrial fission itself is not sufficient to induce mitophagy, for which concomitant mitochondrial damage such as depolarization, increased ROS, misfolded proteins, or other as yet undefined signals are required.

#### PINK1/Parkin-Mediated Mitophagy

PINK1 is a serine/threonine kinase with a mitochondrial target sequence (MTS) and transmembrane domain (TMD), while Parkin is a cytosol ubiquitin E3 ligase. Both PINK1 and Parkin are primarily associated with Parkinson’s disease (Kitada et al., 1998; Valente et al., 2004) and are the most recognized proteins that regulate mitophagy for mitochondrial maintenance and quality control (Pickles et al., 2018). In healthy mitochondria, PINK1 is constitutively transported into the IMM by the TOM/TIM complex in a mitochondrial membrane potential (ΔΨ_m_)-dependent manner (Becker et al., 2012; Sekine and Youle, 2018), where the MTS is cleaved by the mitochondrial processing peptidase (MPP), and TMD is cleaved by presenilin-associated rhomboid-like protease (PARL; Shi and McQuibban, 2017; Sekine and Youle, 2018). The remaining 52kDa fragment, which harbors the kinase domain of PINK1, is exposed to the cytosol until its proteasomal degradation (Sekine and Youle, 2018).

In damaged mitochondria, upon ΔΨ_m_ depolarization, PINK1 importation is prevented, leading to the accumulation of full-length PINK1 on the OMM (Harper et al., 2018; Sekine and Youle, 2018). However, it has also been shown that the excessive misfolded proteins in the mitochondrial matrix alone are capable of inducing PINK1 accumulation, regardless of ΔΨm loss (Jin and Youle, 2013), suggesting that ΔΨ_m_ depolarization is not a prerequisite for PINK1 accumulation. PINK1 kinase is activated through auto-phosphorylation (Okatsu et al., 2012; Harper et al., 2018), and subsequently phosphorylates ubiquitin at Ser65 (Wauer et al., 2015), which triggers recruitment and activation of Parkin (Ordureau et al., 2014; Wauer et al., 2015). Once activated on mitochondria, Parkin drives a feed-forward program of mitochondrial ubiquitination that generates poly-ubiquitin chains, which are substrates for PINK1 and function as “eat-me” signals for damaged mitochondria, thereby amplifying mitophagy (Ordureau et al., 2014; Lazarou et al., 2015). A majority of these findings are performed under artificially prepared experimental conditions using immortal cell lines and RNA interference-based *Pink1* or *Parkin* silencing technique, thereby making it difficult to analyze the molecular signaling of this feed-forward process in a physiological context. Indeed, it has been shown that endogenous Parkin is not sufficient to induce mitophagy in human primary fibroblasts and induced pluripotent stem-derived neurons upon loss of the ΔΨm (Rakovic et al., 2013).

Many studies have contributed to delineating how ubiquitinated mitochondria are delivered to the autophagy machinery. Following Parkin-mediated ubiquitination of mitochondrial substrates, mitochondria are sequestrated into the isolation membrane by interacting with adaptor proteins [such as p62, optineurin (OPTN), NDP52, TAX1BP1, and NBR1] on the isolation membrane (Lazarou et al., 2015). Similar to the mechanism of general autophagy, microtubule-associated protein 1 light chain 3 (LC3) recognizes and interacts with these adaptor molecules through LC3-interacting region (LIR) motifs, and subsequently initiates mitophagosomal formation (Lazarou et al., 2015). It has been demonstrated that p62 binds ubiquitin chains on depolarized mitochondria and is essential for mitochondrial clustering and subsequent mitochondrial elimination in acute myeloid leukemia *in vitro* and *in vivo* (Nguyen et al., 2019). However, Lazarou et al. (2015) suggested that p62 is dispensable for the elimination of damaged mitochondria in antimycin A-treated HeLa cells. Mechanically, antimycin A activates PINK1 to induce mitophagy through the phospho-ubiquitin-mediated recruitment of NDP52 and OPTN, independently of p62. These controversial findings challenge the role of p62 in the PINK1/Parkin pathway, prompting us to investigate further. OPTN, another autophagy adaptor with a ubiquitin-binding domain and an LIR motif, is recruited to damaged mitochondria *via* association with ubiquitinated OMM proteins in a Parkin-dependent manner, and subsequently induces sequestration of mitochondria by autophagosomes by interacting with LC3 (Wong and Holzbaur, 2014). OPTN was recently identified as being phosphorylated by the serine/threonine-protein kinase TANK-binding kinase 1 (TBK1), which is activated by mitochondrial depolarization in HeLa cells (Richter et al., 2016). The OPTN–TBK1 complex formation establishes a feed-forward mechanism in mitochondrial clearance. A question remains as to whether TBK1 kinase activity would have an impact on the other proteins, other than autophagy adaptors, in the maintenance of mitochondrial homeostasis.

#### Receptor-Mediated Mitophagy

Mitophagy receptors, containing LIR motif and commonly localized in the OMM and IMM, can directly induce mitophagy. Certain OMM mitophagy receptors, including FUN14 domain-containing protein 1 (FUNDC1; Chen et al., 2014), BNIP3 and BCL2 interacting protein 3 like (BNIP3L/NIX; Lee et al., 2017), and FKBP prolyl isomerase 8 (FKBP8; Bhujabal et al., 2017), have recently been identified to promote mitophagy. Prohibitin 2 (PHB2; Wei et al., 2017; Yan et al., 2019b) and cardiolipin (CL; Chu et al., 2013; Kagan et al., 2016) have been identified as novel IMM mitophagy receptors. Receptor-mediated mitophagy is regulated at the transcriptional or post-transcriptional level in response to different mitochondrial stresses (Palikaras et al., 2018).

FUNDC1 is a conserved mitophagy receptor associated with the hypoxia-induced mitophagy. Under normoxic conditions, FUNDC1 is phosphorylated at Tyr18 by SRC kinase (Liu et al., 2012) and at Ser13 by CK2 kinase (Chen et al., 2014), which suppresses the activity of the FUNDC1 LIR motif for subsequent mitophagy (Kuang et al., 2016). Under hypoxic conditions, the mitochondrial phosphatase PGAM5 dephosphorylates FUNDC1, disrupting the physical association between FUNDC1 and OPA1 and then inhibiting mitochondrial fusion (Chen et al., 2014, 2016). In turn, FUNDC1 translocates to the MAM, mediating Drp1 recruitment and promoting mitochondrial fragmentation (Wu et al., 2016; Ma et al., 2019). The FUNDC1 LIR motif is also activated and binds to LC3, initiating mitophagy (Liu et al., 2012). Hence, FUNDC1 coordinates mitochondrial morphology and mitophagy in response to stress. Simultaneously, the serine/threonine-protein kinase Unc-51-like kinase 1 (ULK1) is upregulated and translocated to damaged mitochondria, where it phosphorylates FUNDC1 at Ser17, triggering mitophagy (Wu et al., 2014). A recent study suggested mitochondrial E3-ubiquitin protein ligase 5 (MARCH5) specifically mediates FUNDC1 ubiquitylation at Lys119 to promote its proteasomal degradation and prevent aberrant mitophagy, avoiding improper clearance of undamaged mitochondria under hypoxic stress (Chen et al., 2017). Hence, the MARCH5/FUNDC1 axis may function as a negative feedback mechanism to protect undamaged mitochondria from excessive mitophagic removal.

BNIP3/NIX are atypical members of the pro-apoptotic BCL2 family and contain an atypical BH3 domain (Yasuda et al., 1998; Imazu et al., 1999). Under hypoxic stress, BNIP3 is upregulated by hypoxia-inducible factor 1 (HIF-1), initiating LC3-dependent mitophagy and precluding mtROS overproduction (Lee et al., 2017). BNIP3 phosphorylation at Ser17 and Ser24 (Zhu et al., 2013) and NIX phosphorylation at Ser34 and Ser35 (Rogov et al., 2017) enhance their association with LC3, in turn promoting NIX/BNIP3-mediated mitophagy. BNIP3 is indicated to promote mitophagy by suppressing PINK1 cleavage (Zhang et al., 2016), while NIX-mediated mitophagy is unaffected by *Pink1* knockdown (Gao et al., 2015; McWilliams and Muqit, 2017), suggesting that different mechanisms exist in the regulation of BNIP3‐ or NIX-mediated mitophagy. Both NIX and BNIP3 have been implicated in mitochondrial Parkin translocation (Ding et al., 2010; Lee et al., 2011), and NIX is then ubiquitinated by Parkin enzymatic activity, which promotes its recognition by autophagy adaptors and enhances mitophagy in the PINK1/Parkin pathway (Gao et al., 2015). Therefore, interaction between the mitophagy receptor and PINK1/Parkin-mediated mitophagy requires further investigation in cell type‐ and tissue-specific contexts.

FKBP8 (also known as FKBP38) is a novel OMM mitophagy receptor interacting with LC3 to initiate mitochondrial degradation in a Parkin-independent manner (Bhujabal et al., 2017). Interestingly, FKBP8 escapes from mitophagic degradation *via* translocation from the mitochondria to the ER during Parkin-mediated mitophagy induced by the mitochondrial uncoupler carbonyl cyanide 3-chlorophenylhydrazone (CCCP) *in vivo*, which is essential for suppression of unwanted apoptosis (Saita et al., 2013; Bhujabal et al., 2017). However, under physiological conditions or pressure overload, no effects on mitophagy were observed in FKBP8-deficient HEK293 cells or H9c2 myocytes (Misaka et al., 2018). Thus, how FKBP8 is activated as a mitophagy receptor and the underlying mechanism responsible for its translocation warrant further investigation.

PHB2, an IMM scaffold protein, has been identified as a putative mitophagy receptor under stress conditions. Upon mitochondrial membrane depolarization or misfolded protein aggregation, PHB2 mediates stabilization of PINK1 on the OMM and promotes mitochondrial recruitment of Parkin by negatively modulating PARL and protecting PGAM5 from cleavage (Yan et al., 2019b), leading to proteasome-dependent mitochondrial OMM rupture (Wei et al., 2017; Yan et al., 2019b). PHB2 is then externalized to the OMM and interacts with LC3, leading to phagophore generation and subsequent elimination of dysfunctional mitochondria, preserving cellular homeostasis (Wei et al., 2017; Xiao et al., 2018). Yan et al. (2019b) reported that PHB2 with mutations of the LIR motif also significantly promotes accumulation of PINK1 and subsequent recruitment of Parkin to the mitochondria, suggesting that PHB2 regulates PINK1/Parkin-mediated mitophagy independent of interacting with LC3.

CL is a phospholipid distributed along the IMM of healthy mitochondria to support cristae. Recent studies have emphasized the critical role of CL in mitophagy. Kagan et al. (2016) demonstrated that NDPK-D, a hexameric intermembrane space protein, can interact with CL to facilitate its translocation to the OMM during mitochondrial damage. Chu et al. (2013) identified that CL is externalized to the OMM in several types of cells, serving as an “eat-me” signal for the elimination of damaged mitochondria. Once externalized, CL directly binds to LC3 for mitophagy induction (Chu et al., 2013; Kagan et al., 2016). Given that oxidized CL can be released from mitochondria to contribute to apoptosome formation (Kagan et al., 2005), and mitophagic degradation of externalized CL could suppress activation of mitochondrial death pathways.

### Physiological and Pathological Effects of Mitophagy

**Figure 2:**
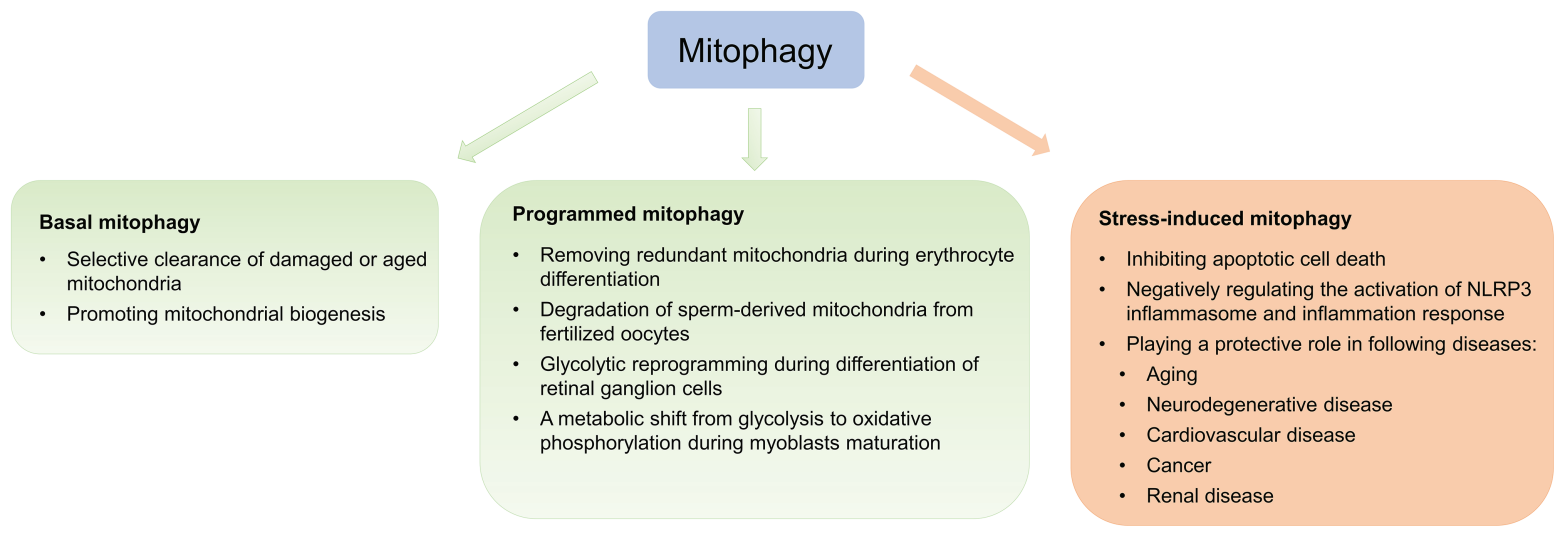
Physiological and pathological effects of mitophagy. Mitophagy is classified as basal, programmed, or stress-induced. Basal mitophagy is essential for routine mitochondrial maintenance. Programmed mitophagy removes redundant mitochondria during differentiation and development. Stress-induced mitophagy occurs under pathological context.

As mentioned above, complex interplay among multiple mitophagy pathways ensures energy metabolism and tissue homeostasis. According to physiological and pathological contexts, mitophagy is classified as basal, programmed, or stress-induced (Figure 2; Palikaras et al., 2018).

Basal mitophagy, referred to as under steady-state mitophagy levels, is a continuous process under which aging and damaged mitochondria are selectively eliminated and recycled (Sun et al., 2015; McWilliams et al., 2016). Recently, two transgenic mouse models expressing mitochondria-targeted pH-sensitive fluorescent reporters have been generated to analyze basal mitophagy, revealing that basal mitophagy levels vary between cell types during routine mitochondrial maintenance (Sun et al., 2015; McWilliams et al., 2016). Basal mitophagy exhibits tissue-specific distribution, with low levels in the thymus and high levels in the heart and kidney, which are energetically demanding tissues (Sun et al., 2015).

Crosstalk between the mitophagy pathway and the mitochondrial biogenesis pathway has been identified (Palikaras et al., 2015). Given that mitochondrial biogenesis is preceded by mitophagy (Sin et al., 2016), Shin et al. (2011) identified PARIS as a new Parkin-interacting substrate, whose levels are regulated by the ubiquitin proteasome system (UPS) *via* interaction with Parkin. PARIS regulates mitochondrial biogenesis by transcriptionally repressing PGC-1α (Stevens et al., 2015). Upon activation of mitophagy, Parkin ubiquitinates PARIS and promotes its degradation, increasing transcription of PGC-1α and promoting mitochondrial biogenesis (Shin et al., 2011). Parkin inactivation contributes to PARIS accumulation, resulting in chronic repression of PGC-1α and inhibition of mitochondrial biogenesis (Stevens et al., 2015). Thus, mitophagy can regulate mitochondrial quality and quantity to sustain energy metabolism by regulating mitochondrial biogenesis. Surprisingly, McWilliams et al. (2018) discovered that endogenous PINK1 is dispensable for basal mitophagy in mouse tissues with high metabolic demand, in spite of detectable PINK1 under physiological states. *Drosophila* with *Pink1*/*parkin* mutations also exhibits detectable and abundant basal mitophagy in many tissues (Lee et al., 2018). These studies suggest that PINK1 and Parkin are dispensable and not the major drivers of basal mitophagy. Mechanisms responsible for basal mitophagy are yet fuzzy. In light of this, the potential role of another compensatory mechanism for mitophagy promotion of mitochondrial biogenesis will be the topic of future studies.

During differentiation and development, the primary role of programmed mitophagy is to remove redundant mitochondria. The most representative example is NIX, which functions as a mitochondrial receptor to mediate mitochondrial removal through mitophagy during erythrocyte differentiation (Sandoval et al., 2008). *Nix* ^−/−^ mice develop anemia secondary to defective mitochondrial elimination (Sandoval et al., 2008). Additionally, in coordination with UPS, programmed mitophagy regulates degradation of sperm-derived mitochondria from fertilized oocytes dependent of canonical autophagy receptors LC3 in *Caenorhabditis elegans* (Al Rawi et al., 2011; Sato and Sato, 2011), preventing inheritance of paternal mtDNA (Yan et al., 2019a). Yet, LC3 and gamma-aminobutyric acid receptor-associated protein are dispensable during mammalian sperm mitophagy (Song et al., 2016a), suggesting sperm mitophagy in mammals and other taxa involves a non-canonical ubiquitin-recognizing autophagic pathway independent of LC3. A recent study suggests that *Pink1* or *Parkin* in combination with *MUL1* is required for paternal mitochondrial elimination in mammals (Rojansky et al., 2016). Therefore, it appears that the involvement of sperm mitophagy in post-fertilization should be investigated in other, higher mammalian models. Physiological functions, such as energy metabolism and gene expression, are also altered during the cell differentiation process, which involves cellular reprogramming. Esteban-Martinez et al. (2017) observed that the differentiation of retinal ganglion cell is regulated by NIX-mediated mitophagy, leading to glycolytic reprogramming. Contrastingly, during differentiation, myoblasts undergo a metabolic shift from glycolysis to oxidative phosphorylation (Pääsuke et al., 2016). Sin et al. (2016) established that during early myogenic differentiation, p62-mediated mitophagy eliminates mitochondria, followed by Drp1-mediated mitochondrial fragmentation, while mitochondrial biogenesis is promoted during the later phase of myogenic differentiation, leading to the regeneration of a dense mitochondrial network.

Mitophagy is potently induced during stress and facilitates mitochondrial quality control to mediate metabolic adjustments to external challenges. Concurrently, impairment of mitophagy is responsible for mitochondrial dysfunction and progressive accumulation of defective organelles, leading to cell death and tissue damage. Mild and transient oxidative stress induced by H_2_O_2_ and rotenone at low concentrations for short incubation time leads to generation of low ROS levels to specifically induce mitophagy (Frank et al., 2012), suggesting that mitophagy functions as an early cytoprotective response that favors adaptation to stress by removing damaged mitochondria (Kubli and Gustafsson, 2012). Once cellular mitochondria damaged by increased oxidative stress and apoptotic proteases exceed the range that can be removed by mitophagy, programmed apoptotic cell death pathway is then activated (Kubli and Gustafsson, 2012). Additionally, sustained activation of apoptosis leads to caspase-mediated cleavage of Beclin1, mitochondrial stress and subsequent suppression of autophagy in growth factor IL-3-deprived bone-marrow-derived pro-B-cell line (Ba/F3 cells; Wirawan et al., 2010). Therefore, the balance between mitophagy and apoptosis plays a critical role in determining cellular fate under stress conditions, such as hypoxia, oxidative stress, DNA damage, and loss of growth factors (Kubli and Gustafsson, 2012).

Recent studies have also revealed that mitophagy is also associated with the inflammation response initiated by NLRP3 inflammasome activation (Gkikas et al., 2018; Han et al., 2018). Emerging evidence suggests that damaged mitochondria are responsible for NLRP3 activation in disease conditions, due to release of mtDNA, mtROS, CL externalization, and other triggers (Gurung et al., 2015; Gkikas et al., 2018). Blockade of mitophagy leads to the accumulation of damaged, ROS-generating mitochondria, in turn activating the NLRP3 inflammasome (Zhou et al., 2011). Indeed, Kim et al. (2016) demonstrated that SESN2 activates mitophagy to protect against sepsis, maintaining immunological homeostasis. Mitophagy is thus emerging as a critical regulator of the inflammation response, and is therefore a novel putative therapeutic target for inflammatory disease.

## Mitophagy and Renal Diseases

### Mitophagy and AKI

AKI is a clinical syndrome characterized by abrupt loss of renal function and involves sublethal and lethal injury of renal tubule epithelial cells (RTECs; Linkermann et al., 2014). Renal ischemia-reperfusion (IR), nephrotoxins, and sepsis contribute to the pathogenesis of AKI (Havasi and Borkan, 2011; Mehta et al., 2016). Abundant evidence suggests that mitochondrial damage and dysfunction contribute to AKI, especially in the context of RTEC injury and death (Linkermann et al., 2014), manifesting as mitochondrial fragmentation and functional decline in RTECs (Brooks et al., 2009; Hall et al., 2013; Tang et al., 2018; Plotnikov et al., 2019). Brooks et al. (2009) demonstrated that marked mitochondrial fragmentation appears in proximal tubular cells exposed to cisplatin or IR prior to both cytoplasmic cytochrome c release and apoptosis, indicating that this is an important early event in the development of AKI. Hence, therapeutic strategies targeting mitoprotection could be effective in AKI. Compounds that protect mitochondrial integrity, such as mitochondria-targeted antioxidants (Plotnikov et al., 2019), CL-targeted peptides that protect cristae structure (Li et al., 2016), and MA-5 that facilitates ATP production and reduces the level of mtROS (Suzuki et al., 2016), are protective in AKI.

**Figure 3:**
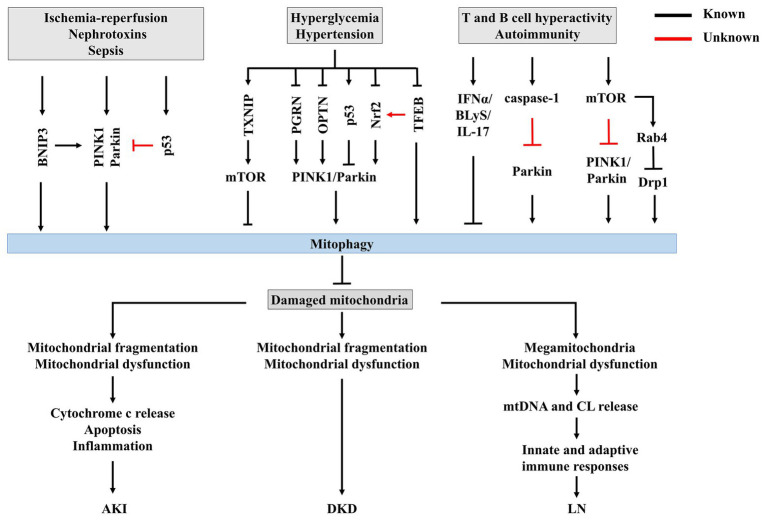
The potential mechanisms of mitophagy in renal disease. Insufficient or impaired mitophagy contributes to an accumulation of damaged mitochondria in kidney, which plays a core role in the progression of acute kidney injury (AKI), diabetic kidney disease (DKD), and lupus nephritis (LN).

Recent work has suggested that mitophagy is involved in the pathophysiological processes of AKI (Figure 3). Tang et al. (2018) demonstrated both PINK1 and Parkin are upregulated in RTECs during ischemic AKI *in vitro* and *in vivo*, while PINK1 and/or Parkin deficiency results in increased mitochondrial damage, ROS production, and inflammation in this context. Together, these pathologies result in increased tubular damage and aggravated AKI, supporting a protective role for PINK1/Parkin-mediated mitophagy in mitochondrial quality control in the context of tubular cell survival and function in AKI. Indeed, BNIP3 is also upregulated in Boston University mouse proximal tubular cell line (BUPMT cells) following oxygen-glucose deprivation-reperfusion, and in kidney tissues of mouse models of IR (Tang et al., 2019). Similarly, BNIP3-deficient mice are susceptible to IR injury, manifested as renal accumulation of damaged mitochondria, increased ROS production, enhanced cell apoptosis, and inflammation, supporting a critical role of BNIP3-mediated mitophagy in IR-induced AKI (Tang et al., 2019). Taken together, these findings strongly support the involvement of multiple mitophagy regulatory pathways in the pathogenesis of AKI.

Lee et al. (2011) demonstrated that overexpression of BNIP3 promotes mitochondrial translocation of Parkin and thus activates mitophagy in cardiac myocytes, while BNIP3-mediated mitophagy is reduced in Parkin-deficient myocytes under physiological condition. In addition, Zhang et al. (2016) reported that BNIP3 suppresses proteolytic cleavage of PINK1 by direct interaction, promoting the accumulation of PINK1 on the OMM, increasing mitochondrial recruitment of Parkin, and the subsequent activation of mitophagy in both HEK293 cells and mouse embryonic fibroblasts, which is consistent with the observation obtained in CCCP-treated HEK293 cells. These studies together suggest that BNIP3 plays a vital role in PINK1/Parkin-mediated mitophagy, and functional upregulation of BNIP3 represents a novel therapeutic strategy to suppress disease progression. Thus, it will be worthwhile to delineate how BNIP3‐ and PINK1/Parkin-mediated mitophagy coordinate to regulate RTEC mitophagy in the context of AKI. This could provide critical insights in regarding mitophagy modulation as a therapeutic intervention for AKI. Likewise, Wang et al. (2020a) unveiled that Bax inhibitor-1 (BI1) promotes mitochondrial retention of PHB2 and improves mitophagy, preserving mitochondrial homeostasis in a murine AKI model. As previous studies demonstrated, PHB2 mediates mitophagy once it is externalized to the OMM (Wei et al., 2017; Xiao et al., 2018), this finding indicates that BI1-PHB2 interaction may prevent PHB2 from exporting into cytoplasm, representing a novel mechanism for mediating mitophagy by mitochondria-localized PHB2, rather than total PHB2. However, the exact mechanisms underlying PHB2 disassociation from mitochondria and BI1 downregulation in AKI have not been identified.

Cisplatin is a widely used and highly effective chemo-therapeutic agent. However, cisplatin treatment causes severe side effects, the most common of which is nephrotoxicity, limiting its therapeutic application (Volarevic et al., 2019). Recently, Wang et al. (2018) demonstrated that renal functional loss, tissue damage, and apoptosis are aggravated in cisplatin-treated *Pink1* ^−/−^ and *Parkin* ^−/−^ mice relative to cisplatin-treated wild-type mice, suggesting that activation of PINK1/Parkin-mediated mitophagy plays a protective role against cisplatin nephrotoxicity. Further, cytosolic p53 interacts directly with Parkin and disrupts its translocation to damaged mitochondria and subsequent clearance of damaged mitochondria by mitophagy in the mouse heart exposed to doxorubicin toxicity (Hoshino et al., 2013). Hence, negative regulation of p53 may promote mitophagy and represent a novel approach to maintain mitochondrial homeostasis. It has been suggested that mitochondrial dysfunction and p53 activation contribute to cisplatin-induced AKI (Wang et al., 2006). Antimycin A and myxothiazol, inhibitors of the mitochondrial respiration complex, ameliorate cisplatin-induced p53 activation and exert cytoprotective effects in cultured RTECs (Wang et al., 2006). Whether antimycin A and myxothiazol induce mitophagy by promoting Parkin activity through p53 downregulation in RTECs remains unclear, and should be investigated further. A recent study by Zhu et al. (2020) demonstrated that trehalose administration attenuates mitochondrial dysfunction through activating transcriptional factor EB (TFEB)-mediated autophagy and mitophagy in cisplatin-induced AKI *in vitro* and *in vivo*. The study sheds lights on the roles of TFEB on mitophagy and provides a novel promising therapeutic target for AKI.

### Mitophagy and DKD

DKD is one of the most serious diabetic microvascular complications and is the major cause of chronic kidney disease (CKD) and end-stage renal disease (ESRD). ESRD is primarily characterized by persistent and increasing leakage of protein (albuminuria) and/or progressive decline in glomerular filtration rate that may end in complete loss of renal function (National Kidney Foundation, 2012). Hyperglycemia and hypertension are two of the primary DKD risk factors (Alicic et al., 2017). The development and progression of DKD have not been fully elucidated. Although control of blood pressure and hyperglycemia decreases the risk of DKD, no presently available therapeutic modalities can delay or prevent the progression of DKD (Alicic et al., 2017).

Recent studies have found that impeded mitophagy is a key pathological mechanism for the development and progression of DKD (Figure 3). Renal function depends on interplay between multiple cell types, including endothelial cells, podocytes, mesangial cells, and tubulointerstitial cells, and is energetically demanding and relying on mitochondrial function (Maezawa et al., 2015). The importance of podocyte injury in the initiation and progression of DKD has been established over the past few decades (Ilatovskaya et al., 2015). However, recent findings have highlighted the role of tubulointerstitial cells in the pathogenesis of DKD (Maezawa et al., 2015; Coughlan et al., 2016). Coughlan et al. (2016) suggested that RTEC injury precedes glomerular damage in a rat model of diabetes, indicating that RTECs are primary initiators of DKD.

Most importantly, within RTECs or glomeruli, mitochondrial dysfunction precedes renal dysfunction in diabetes (Coughlan et al., 2016), strongly supporting the role of mitochondrial dysfunction in DKD (Higgins and Coughlan, 2014; Forbes and Thorburn, 2018; Saxena et al., 2019). Increasing evidence supports that impairment of mitophagy leads to mitochondrial dysfunction, accelerating the progression of DKD. Progressive accumulation of fragmented and swelling mitochondria is observed in diabetic kidneys in both humans and animals (Higgins and Coughlan, 2014). Further, accumulation of mitophagosomes has been detected in renal biopsies from patients with DKD (Chen et al., 2018), and in the kidneys of diabetic mice (Xiao et al., 2017; Zhou et al., 2019). PINK1 and Parkin, two important regulators of mitophagosome formation (Pickles et al., 2018), are significantly decreased in the tubules of diabetic mice (Xiao et al., 2017) and *in vitro* under high-glucose conditions (Xiao et al., 2017; Chen et al., 2018), leading to impairment of mitochondrial turnover. Taken together, these findings support impairment of mitophagy during DKD. Surprisingly, in streptozotocin-induced diabetic rat models, renal cortex PINK1 expression is increased in early diabetes (Smith et al., 2012), indicating that mitophagy could be activated to clear dysfunctional mitochondria from the kidney during early-stage diabetes, but becomes overwhelmed as DKD progresses, resulting in accumulation of fragmented mitochondria and induction of cell death (Higgins and Coughlan, 2014).

Huang et al. (2016) demonstrated that thioredoxin-interacting protein (TXNIP)-dependent activation of the mammalian target of rapamycin (mTOR) signaling pathway contributes to dysfunctional mitophagy in the diabetic kidney. Other studies showed that decreased PINK1 and Parkin expression in DKD occur following deficiency of progranulin (PGRN; Zhou et al., 2019) and OPTN (Chen et al., 2018). These findings suggest that kidney-targeted inhibition of TXNIP or activation of PGRN and OPTN could be novel therapeutic strategies for the prevention and treatment of DKD. Some agents effectively increase mitophagy in experimental DKD. For example, metformin induces mitophagy by promoting Parkin activity through p53 downregulation (Song et al., 2016b). MitoQ, a mitochondrially-targeted antioxidant that has been reported to protect against DKD by suppressing mitochondrial ROS-TXNIP/NLRP3/IL-1β axis activation (Han et al., 2018), was recently discovered to suppress tubular injury *via* nuclear factor-erythroid-2-related factor 2 (Nrf2)/PINK1-mediated mitophagy in experimental DKD (Xiao et al., 2017). Another mitochondrially-targeted antioxidant CoQ10 also exerts beneficial effects in both *in vivo* and *in vitro* models of DKD *via* mitophagy by stimulating Nrf2 signaling (Sun et al., 2019). These studies highlight the crucial role of Nrf2 in regulating mitophagy in DKD. A recent study by Park et al. (2019) suggests that novel regulation of Nrf2 by TFEB mitigates oxidative stress. Interestingly, the activities of both Nrf2 and TFEB were shown to be downregulated in DKD (Xiao et al., 2017; Zhao et al., 2018). With increasing evidence supporting the role of TFEB during mitophagy (Kim et al., 2018; Tan et al., 2019), the involvement of crosstalk between TFEB and Nrf2 in this process should be further investigated.

Taken together, these studies suggest that mitophagy plays a renoprotective role in DKD. However, the pathogenesis of DKD is complex, and the regulatory mechanisms of mitophagy in this context remain largely unknown, warranting further investigation.

### Mitophagy and LN

LN is the most common severe manifestation of systemic lupus erythematosus (SLE; Liu et al., 2013). Severe LN is at high risk of developing into ESRD and is an important predictor of mortality in SLE patients (Tektonidou et al., 2016). The pathogenesis of LN involves extrarenal and intrarenal pathogenic mechanisms, including aberrant T and B cell signaling, autoantibody production, and deregulated cytokine secretion due to genetic variants and environmental factors (Lech and Anders, 2013).

T cells are critical drivers of autoimmunity and related organ damage (Suarez-Fueyo et al., 2017). In T cells of SLE patients, mitochondrial dysfunction, characterized by increased mitochondrial mass (megamitochondria), mitochondrial hyper-polarization, and ATP depletion, contributes to aberrant activation and enhanced necrosis of T cells (Caza et al., 2012; Leishangthem et al., 2016). Subsequently, the necrotic debris of T cells releases extracellular mitochondria (Maeda and Fadeel, 2014). Mitochondria and their components, such as mtDNA and CL, are recognized as damage-associated molecular patterns (DAMPs) that initiate innate and adaptive immune responses to elicit an inflammatory response that triggers organ damage (Grazioli and Pugin, 2018), suggesting a crucial role of mitochondrial metabolism in immunological fate. Multiple mechanisms contribute to mitochondrial abnormalities in lupus T cells. On one hand, nitric oxide (NO)-dependent mitochondrial biogenesis could account for megamitochondria (Nagy et al., 2004), leading to sustained T cell activation (Ron-Harel et al., 2016). On the other hand, increased T cell mitochondria in SLE have also been attributed to insufficient mitophagy (Figure 3; Perl, 2013). Sequestration and successful clearance of damaged mitochondria by mitophagy suppress mtROS accumulation and prevent inflammation and generation of autoantigens by intracellular oxidation (Minton, 2016), suggesting that mitophagy is a potential therapeutic target for SLE and LN.

Current evidence suggests that impaired mitochondrial degradation could contribute to SLE (Caza et al., 2014; Gkirtzimanaki et al., 2018). Sustained IFNα signaling is linked to the pathogenesis SLE, promoting a pathological axis of BLyS and IL-17 (López et al., 2016). Recently, Gkirtzimanaki et al. (2018) observed that IFNα damages mitochondrial metabolism and mediates lysosomal dysfunction, impeding mitochondrial clearance and leading to cytosolic accumulation of mtDNA in SLE monocytes. In addition, caspase-1, the central component of the NLRP3 inflammasome, is activated in the podocytes of both LN patients and lupus-prone mice (Fu et al., 2017), and was recently demonstrated to inhibit mitophagy and amplify mitochondrial damage, mediated by cleavage of the key mitophagy regulator Parkin in lipopolysaccharide (LPS)-primed bone-marrow-derived macrophages (Yu et al., 2014). However, whether caspase-1 inactivates Parkin and thus impairs mitophagy in LN is still unknown.

A previous study showed that mTOR is activated in SLE, and subsequently enhances expression of Rab4, a small GTPase, in lupus T cells (Fernandez et al., 2009). mTOR hyperactivity represses PINK1 expression and decreases Parkin translocation to mitochondria in tuberous sclerosis complex 2-knockout pancreatic β cells exposed to CCCP, indicating that activation of mTOR could impede mitophagy (Bartolome et al., 2017). Nonetheless, the potential contribution of mTOR hyperactivity to impairment of mitophagy in LN remains elusive.

A recent study revealed that Drp1, which initiates mitophagy following mitochondrial fragmentation and lysosomal degradation of mitochondria (Fonseca et al., 2019), is profoundly reduced in T cells from SLE patients and lupus-prone mice, concomitant with the accumulation of mitochondria (Caza et al., 2014). The loss of Drp1 is independent of mTOR activation, and is instead regulated by Rab4-regulated lysosomal degradation (Caza et al., 2014), challenging the interaction between mTOR and Rab4. Importantly, Rab4 blockade with 3-PEHPC leads to Drp1 restoration and reverses mitochondrial accumulation as well as antinuclear antibody (ANA) production, proteinuria, and LN in lupus-prone mice, identifying negative regulation of Rab4 as a potential target for promoting mitophagy in treatment of SLE (Caza et al., 2014).

Given that multiple signaling pathways are involved in the impairment of mitophagy in SLE and LN (Caza et al., 2014; Fu et al., 2017; Gkirtzimanaki et al., 2018), multi-targeted therapy will be necessary to induce remission and prevent flares of SLE and LN. Rapamycin prevents the development of LN in lupus-prone mice (Lui et al., 2008) and SLE patients (Fernandez and Crow, 2018; Eriksson et al., 2019) by inhibiting mTOR and enhancing autophagosome formation and autolysosomal degradation (Chen and Fang, 2002). Recent studies have underscored the utility of rapamycin in protecting mitochondrial function in the context of SLE. Rapamycin increases Drp1 *via* mTOR inhibition in lupus-prone mice (Oaks et al., 2016) and decreases mitochondrial dysfunction by activating mitophagy (Li et al., 2018).

Accordingly, proficient mitophagy may have therapeutic effects in LN, and drugs that induce mitophagy, such as rapamycin and 3-PEHPC, merit further exploration as therapeutic strategies to enhance the clearance of injury-promoting fragmented mitochondria and accelerate recovery from SLE and LN flares.

## Conclusions and Future Directions

Since Lemasters proposed the term “mitophagy” in 2005, many efforts on molecular mechanisms of mitophagy have been made in physiological and pathological contexts. Several mitophagy signaling pathways, including PINK1/Parkin-mediated mitophagy and receptor-mediated mitophagy, such as FUNDC1, BNIP3/NIX, FKBP8, PHB2, and CL, participate in the regulation of mitochondrial content and metabolism preserving homeostasis. Impairment of mitophagy contributes to the pathogenesis of many renal diseases, including AKI, DKD, and LN. Mitochondrial dysfunction serves as a link between mitophagy and these diseases. However, the detailed regulatory mechanisms of mitophagy during the development of these renal diseases remain largely unclear. Therefore, further investigation is warranted to identify key regulators.

As of today, activation of mitophagy has been shown to delay and attenuate many renal diseases. Hence, pharmacological augmentation of mitophagy represents a promising therapeutic strategy for patients with impaired kidney function. Compounds, including activators of PINK1/Parkin or Nrf2 (e.g., metformin, MitoQ, and CoQ10) and TFEB activator trehalose, have been confirmed to increase mitophagy in experimental DKD or AKI (Song et al., 2016b; Xiao et al., 2017; Sun et al., 2019; Zhu et al., 2020), providing substantial evidence in future clinical research. A recent study reports that dietary supplementation with spermidine conveys cardioprotective effects through activation of mitophagy and ameliorates renal injury *via* the induction of autophagy in salt-induced hypertension rodent models (Eisenberg et al., 2016). As incidence and prevalence associated with hypertension have increased in patients with renal abnormalities, it is worth further exploring the relationship between spermidine-mediated renoprotection and mitophagy.

Mitophagy decreases mitochondrial quantity by eliminating redundant or damaged mitochondria, while mitochondrial biogenesis elevates mitochondrial mass to meet higher energy demand and/or to replace the mitochondria that have been removed by mitophagy; therefore, administration of therapeutic mitophagy inducer alone to patients should be prudent. Moreover, whether mitophagy can be specifically targeted is still a great challenge currently. Answering these questions will shed light on cellular mitophagy regulation and facilitate the development of novel therapeutic strategies for renal failure.

## Author Contributions

ZZ, KJ, and HW designed the literature search and wrote the article with input from all authors. CY, QP, and SW were involved in planning and supervised the work. ZZ, LY, and ZL drafted the manuscript and designed the figures. KJ aided in interpreting the results and worked on the manuscript. WL and H-fL designed and directed the project. All authors contributed to the article and approved the submitted version.

### Conflict of Interest

The authors declare that the research was conducted in the absence of any commercial or financial relationships that could be construed as a potential conflict of interest.
